# CELIAC DISEASE PRESENTING WITH DUODENAL ULCERATION

**DOI:** 10.51894/001c.122925

**Published:** 2024-08-31

**Authors:** Mark Rigby

**Affiliations:** 1 Internal Medicine Ascension Genesys

## 50

### INTRODUCTION

Celiac disease (CD) is a rare, gluten-mediated autoimmune disease with an approximate 1% global prevalence. Here, we discuss the case of a 24-year-old female diagnosed with CD after serologic evaluation and biopsy of rare superficial duodenal ulcerations masquerading as inflammatory bowel disease.

### CASE DESCRIPTION

A 24-year-old female with a family history of ulcerative colitis presented with two months of non-bloody, non-bilious emesis. She had associated epigastric and right upper quadrant (RUQ) pain with radiation to the right shoulder, and 30-pound, unintentional weight loss with 7-8 episodes of watery diarrhea per day. She denied previous endoscopy or NSAID use. A negative HIDA scan prompted bi-directional endoscopy which revealed esophagitis, gastritis, superficial duodenal ulcers, with no evidence of colon inflammation. Fecal calprotectin, celiac serology, and fasting gastrin levels revealed elevated tTG-IgA with normal total IgA levels, consistent with celiac sprue. Duodenal biopsy pathology revealed villous attenuation and increased intraepithelial lymphocytes.

### DISCUSSION

Classic signs and symptoms of CD include steatorrhea, weight loss, anemia, and dermatitis herpetiformis. The clinical presentation of this patient was unusual in multiple ways. Nausea and vomiting with RUQ and epigastric abdominal pain with radiation to the right shoulder seemed classic for a biliary origin of disease. The patient’s diarrhea, abdominal pain, weight loss, anemia and family history of ulcerative colitis was suspicious for inflammatory bowel disease. It is rare to find duodenal ulcers at the time of diagnosis of CD. If ulceration occurs, it is more common in an advanced course of the disease and is typically found more distally in the small bowel or colon. Our patient presented with duodenitis and multiple superficial ulcers extending from the duodenal bulb to the second portion of the duodenum. Biopsies remarkable for villous attenuation and increased intraepithelial lymphocytes were characteristic of CD. Both serologic testing and endoscopy with biopsy help aid in diagnosis. tTG-IgA antibody is the preferred test for detection of celiac disease in adults. Adherence to a gluten free diet is pertinent for disease remission.

### CONCLUSION

Clinicians should be aware of atypical CD presentation. Failure to recognize atypical presentations may lead to increased morbidity, mortality and health expenditure.

